# Multi-omics analysis reveals gut microbiota-ovary axis contributed to the follicular development difference between Meishan and Landrace × Yorkshire sows

**DOI:** 10.1186/s40104-023-00865-w

**Published:** 2023-05-01

**Authors:** Baoyang Xu, Wenxia Qin, Yuwen Chen, Yimei Tang, Shuyi Zhou, Juncheng Huang, Libao Ma, Xianghua Yan

**Affiliations:** 1 State Key Laboratory of Agricultural Microbiology, Hubei Hongshan Laboratory, Frontiers Science Center for Animal Breeding and Sustainable Production, College of Animal Sciences and Technology, Huazhong Agricultural University, Wuhan, 430070 Hubei China; 2 The Cooperative Innovation Center for Sustainable Pig Production, Wuhan, 430070 Hubei China; 3 Hubei Provincial Engineering Laboratory for Pig Precision Feeding and Feed Safety, Wuhan, 430070 Hubei China

**Keywords:** Follicular development, Granulosa cells apoptosis, Gut microbiota, Short-chain fatty acids, Sows

## Abstract

**Background:**

The mechanism by which Meishan (MS) sows are superior to white crossbred sows in ovarian follicle development remains unclear. Given gut microbiota could regulate female ovarian function and reproductive capacity, this study aimed to determine the role of gut microbiota-ovary axis on follicular development in sows.

**Methods:**

We compared the ovarian follicular development, gut microbiota, plasma metabolome, and follicular fluid metabolome between MS and Landrace × Yorkshire (L × Y) sows. A H_2_O_2_-induced cell apoptosis model was used to evaluate the effects of multi-omics identified metabolites on the apoptosis of porcine ovarian granulosa cells in vitro.

**Results:**

Compared with L × Y sows, MS sows have greater ovary weight and improved follicular development, including the greater counts of large follicles of diameter ≥ 5 mm, secondary follicles, and antral follicles, but lesser atretic follicles. The ovarian granulosa cells in MS sows had alleviated apoptosis, which was indicated by the increased BCL-2, decreased caspases-3, and decreased cleaved caspases-3 than in L × Y sows. The ovarian follicular fluid of MS sows had higher concentrations of estradiol, progesterone, follicle-stimulating hormone, luteinizing hormone, and insulin like growth factor 1 than L × Y sows. Gut microbiota of MS sows formed a distinct cluster and had improved alpha diversity, including increased Shannon and decreased Simpson than those of L × Y sows. Corresponding to the enhanced function of carbohydrate metabolism and elevated short-chain fatty acids (SCFAs) in feces, the differential metabolites in plasma between MS and L × Y sows are also mainly enriched in pathways of fatty acid metabolism. There were significant correlations among SCFAs with follicular development, ovarian granulosa cells apoptosis, and follicular fluid hormones, respectively. Noteworthily, compared with L × Y sows, MS sows had higher follicular fluid SCFAs concentrations which could ameliorate H_2_O_2_-induced porcine granulosa cells apoptosis in vitro.

**Conclusion:**

MS sows have more secondary and antral follicles, but fewer atretic follicles and apoptotic ovarian granulosa cells, as well as harbored a distinctive gut microbiota than L × Y sows. Gut microbiota may participate in regulating ovarian follicular development via SCFAs affecting granulosa cells apoptosis in sows.

**Supplementary Information:**

The online version contains supplementary material available at 10.1186/s40104-023-00865-w.

## Background

Ovulation rate, the number of oocytes ovulated from matured ovarian follicles per estrus cycle, is the decisive factor affecting the litter size in sows [1, 2]. More than 99% of the developing ovarian follicles undergo atretic degeneration before ovulation in mammalian [3, 4]. Earlier comparative study showed that high fertility Meishan (MS) sows had a higher number of antral/pre-ovulatory follicles than white crossbred sows [5, 6]. Miller et al. found that follicular fluid estradiol concentrations and total estradiol synthesis by granulosa cells were higher in MS sows than in Large-White hybrid sows, which may account for the superiority of MS sows in follicular development [5]. However, the mechanism by which MS sows maintain a superiority in follicular development during the follicular phase remains unclear.

The gut microbiome, as part of hologenome (the collective genomic content of a host and its microbiome), has reported to affect ovarian function and reproductive capacity [7–11]. For sows, dietary prebiotics and probiotics supplementation could improve reproductive performance in association the gut microbiota [12–14]. Moreover, large-scale comparative research between high- and low-reproductive performance sows from different commercial farms suggested that litter size of sows was likely associated with the compositions of the gut microbiota [15]. This close correlation between litter size and gut microbiota had been also found in MS sows [16]. The decrease in litter size of MS sows induced by commercial feeds could be improved by crude fiber supplementation in association with gut microbiota [16]. Gut microbiota had also been proved to mediate the benefits of dietary inulin and cellulose supplementation on ovarian oocyte mature in sows [17]. Overall, gut microbiota may involve in regulation of the litter size of sows by affecting the ovarian follicular development and subsequent ovulation rate.

Given that MS sows can have 11.3 more pre-ovulatory follicles and 10 more released oocytes in an estrus cycle than Large-White hybrid sows [5], the aim of this study was to investigate the role of gut microbiota-ovary axis on follicular development by comparing ovarian follicular development, gut microbiota, plasma metabolome, and follicular fluid metabolome in MS and L × Y sows under the same feeding and housing conditions.

## Methods

### Experimental design and animals

This animal experiment was carried out in accordance with the recommendations of the Guide for the Care and Use of Laboratory Animals Monitoring Committee of Hubei Province, China, and the protocol was approved by the Scientific Ethics Committee of Huazhong Agricultural University (approval numbers HZAUSW-2018-015).

Eight MS sows (age 95 ± 7 d and weight 26.94 ± 3.48 kg) from the National Conservation Farm of MS Pigs (Jiading, Shanghai) and 7 L × Y sows (age 90 ± 4 d and weight 38.98 ± 4.45 kg) from a commercial farm were housed in the same pig-house and had ad libitum access to water and diets. The diets (Table S1) were prepared according to the National Research Council [18]. At 24 h after detection of the third estrus (the average estrous cycle is 21 d), 7 MS sows and 7 L × Y sows were sacrificed for sampling (one MS sow was not reached puberty and was removed). The blood samples were obtained via anterior vena cava puncture into heparinized vacutainer tubes and then centrifuged (4 °C, 3,500 × *g*, for 10 min) for plasma. Immediately after slaughter, feces from rectum were collected and placed in liquid nitrogen. The weight and large follicle count (diameter ≥ 5 mm) of left and right ovaries were determined, respectively. Half of the left ovary was fixed in 4% paraformaldehyde solution for measuring ovarian morphology and the rest were placed in liquid nitrogen. The contents of follicles (diameter ≥ 3 mm) from the right ovary were pooled and collected in a tube and allowed to settle for 5 min [19]. The supernatant was removed and centrifuged at 1,900 × *g* at 4 °C for 30 min to separate granular cells from the follicular fluid [19]. The follicular fluid and granular cells were stored at −80 °C until further analysis.

### Reagents

Paraformaldehyde (#P6148, Sigma-Aldrich, St. Louis, MO, USA), DMEM (#D6429, Sigma-Aldrich, St. Louis, MO, USA), FBS (#SH30406, HyClone, Logan, Utah, USA), penicillin/streptomycin (#15140122, Gibco, Grand Island, NY, USA), H_2_O_2_ (#323381, Sigma-Aldrich, St. Louis, MO, USA), BCL-2 (#3498S, CST, Danvers, MA, USA), Caspase-3 (#9662S, CST, Danvers, MA, USA), and Cleaved Caspase-3 (#9664S, CST, Danvers, MA, USA).

### Hematoxylin and eosin (H&E) staining

H&E staining was conducted as reported [20]. Briefly, after fixation in 4% paraformaldehyde (#P6148, Sigma-Aldrich, St. Louis, MO, USA) overnight at 4 °C, ovary tissues (*n* = 7) were dehydrated in a graded series of alcohols and then embedded in paraffin wax. The section (10 μm) of embedded ovary tissue was dried at 56 °C for 24 h. Next, slides were rehydrated in a graded series of ethanol. After a brief wash in distilled water, we incubated the slides with hematoxylin solution. The sections were then washed with running tap water to remove excess hematoxylin. Then, we differentiated the sections in 1% acid alcohol for 30 s and then washed them with running tap water for 1 min. This step was followed by an incubation in the eosin counterstain, subsequent dehydration in a graded series of ethanol, and immersion in xylene.

### Ovarian follicle counting

Follicle counting was performed as previously described with minor modifications [21]. Briefly, primordial follicles were characterized as having a compact oocyte surrounded by a single layer of flattened granular cells, while primary follicles were identified by the presence of an enlarged oocyte surrounded by a single layer of cuboidal granular cells. Secondary follicles were defined as having an enlarged oocyte surrounded by at least a partial or complete second layer of cuboidal granular cells but no more than four complete layers of cuboidal granular cells. Antral follicles were characterized by the presence of areas of follicular fluid (antrum) or a single large antral space. Follicles at the primordial, primary, and preantral stages of development were deemed atretic if the oocyte was degenerating (convoluted and condensed or fragmented) or absent. To avoid repeat counting a follicle, the numbers of primordial and primary follicles were counted in the randomly selected ten areas of the same size (4 mm^2^), and the numbers of secondary, antral, and atretic follicles were counted in the whole section of H&E-stained ovary (*n* = 7).

### Culture and treatment of sow granular cells

Fresh sow ovaries (20 selected ovaries from 13 sows at 2–3 estrous cycle) were obtained from a commercial slaughterhouse. Sow ovarian granulosa cells were collected from follicles (about 200 follicles with diameter 3–6 mm in total) using a syringe [22, 23]. The granulosa cells were washed and centrifuged (1,000 × *g*, 5 min), and then inoculated in a 6-cm dish. Cells were cultured in a complete culture medium containing 89% DMEM (#D6429, Sigma-Aldrich, St. Louis, MO, USA), 10% FBS (#SH30406, HyClone, Logan, Utah, USA), and 1% penicillin-streptomycin (#15140122, Gibco, Grand Island, NY, USA) at 37 °C under 5% CO_2_ throughout this trial. When the confluence of the cells reached approximately 90%, the cells were transferred to 12-well plate for the next experiment. H_2_O_2_-induced apoptosis model of sow granulosa cells was constructed as previously described with minor modifications [24]. Briefly, granular cells were pre-treated with SCFAs (acetate 95.72 μmol/L, propionate 8.65 μmol/L, butyrate 12.56 μmol/L, isobutyrate 6.38 μmol/L, valerate 2.21 μmol/L, and isovalerate 14.91 μmol/L) or the vehicle PBS for 4 h. Then, the granulosa cells were exposed to H_2_O_2_ (#323381, Sigma-Aldrich, St. Louis, MO, USA) (200 μmol/L) [24] or the vehicle PBS for another 24 h. Subsequently, we conducted apoptosis analysis of granular cells with the terminal terminal-deoxynucleoitidyl transferase mediated nick end labeling (TUNEL) assay (*n* = 7).

### Apoptosis analysis of granular cells

The TUNEL assay was used to mark apoptotic ovarian granular cells in paraffin section [25]. For paraffin ovary slides (10 μm), after deparaffinize, rehydrate, antigen retrieval, and permeabilization, slides were treated with appropriate amount mix of TDT enzyme, dUTP and buffer (1:5:50) in the tunel kit according to the number of slices and tissue size. These slides were then placed in a flat wet box, incubate at 37 °C for 2 h. Follow DAPI counterstain in nucleus, slides were washed three times with PBS (pH 7.4) in a Rocker device, 5 min each. For granular cells in vitro, after fixation in 4% paraformaldehyde and permeabilization with 0.3% Triton X-100 for 5 min, cells were treated for TUNEL assay in the dark. In the negative control group, recombinant TdT Enzyme was replaced with ddH_2_O. The cells with red fluorescence were defined as apoptotic cells, and the ratio of TUNEL positive granular cells were determined using computer-assisted morphometry (Nikon ECLIPSE Ti2, Tokyo, Japan).

### Western blot analysis

The granular cells were homogenized in RIPA buffer and then on ice for 30 min. After centrifugation at 12,000 × *g*, 4 °C for 15 min, the supernatants were collected, and their protein concentrations were determined using BCA kit (Thermo Scientific, 23250). An equivalent amount of protein (12 μg) was separated by polyacrylamide gel electrophoresis and transferred to a polyvinylidene fluoride membrane. After TBST cleaning and sealing with 5% skim milk, the membrane was incubated with rabbit polyclonal antibodies BCL-2 (CST, #3498S, 1:1,000), Caspase-3 (CST, #9662S, 1:1,000), and Cleaved Caspase-3 (CST, #9664S, 1:1,000) at 4 °C overnight. The membrane was then incubated with secondary antibody (1:10,000) at room temperature for 1.5 h. Bands were measured by densitometry using image J software and relative protein expression levels were standardized with β-actin.

### Quantitative enzyme-linked immunosorbent assay (ELISA)

The concentrations of follicle-stimulating hormone (FSH, #CSB-E06791p, sensitivity 10 mIU/mL, and inter/intra assays CV < 15%), estradiol (#CSB-E06844p, sensitivity 40 pg/mL, and inter/intra assays CV < 15%), progesterone(#CSB-E12869p, sensitivity 0.2 ng/mL, and inter/intra assays CV < 15%), and insulin-like growth factor 1(IGF-1, #CSB-E06829p, sensitivity 1.56 ng/mL, intra assays CV < 8%, and inter assays CV < 10%) in follicular fluid were determined using commercial pig ELISA kits (Cusabio, Wuhan, China) according to the manufacturer’s instructions.

### Gut microbiota profiling

After thoroughly homogenizing with zirconia beads, the total genomic DNA of a single sow’s fecal microbiota was extracted using the TGuide S96 Magnetic Soil/Stool DNA kit (TIANGEN, Beijing, China) according to manufacturer’s instructions. Concentration and purity of the DNA were determined using a NanoDrop ND-1000 spectrophotometer (NanoDrop Technologies, Rockland, DE, USA). V4 hypervariable regions of 16S rRNA genes (515F, 5′-GTGYCAGCMGCCGCGGTAA-3′; 806R2, 5′-GGACTACNVGGGTWTCTAAT-3′) was used for PCR amplification. Purified amplicon products were pooled in equimolar and paired end sequenced (2 × 250) on an Novaseq 6000 platform (Illumina, San Diego, USA) at Biomarker Technologies Co., Ltd. (Beijing, China).

The sequencing raw data was analyzed using the QIIME2 platform (version 2021.6) [26]. USEARCH [27] (version 10.0) was employed to cluster sequences into operational taxonomic units (OTUs) with similarity of over 97%. The taxonomy of each OTU representative sequence was determined by RDP Classifier against the 16S rRNA gene database Silva [28] (Release132) and Greengenes [29] (version 13.5). Principal co-ordinates analysis (PCoA) analysis and permutational multivariate analysis of variance (PERMANOVA, with 999 Monte Carlo permutations) were conducted based on Bray–Curtis distances using the package “vegan” in R software (version 4.3.1) [30]. The different genera were identified using linear discriminant analysis (LDA) effect size (LEfSe) analysis [31].

### Analysis of gut microbial function and metabolites

The function of gut microbiota was analyzed using PICRUSt2 based on 16S rDNA data and reference genome [32]. The gut microbial metabolites short-chain fatty acid (SCFAs) of acetate, propionate, butyrate, valerate, isobutyrate, and isovalerate in rectal feces were determined using gas chromatography with a modification of the previous method [20]. In brief, 1 g of feces samples was weighed into a 2-mL centrifuge tube with 1 mL of methanol added. After being vortexed for 30 s, the sample was centrifuged (12,000 × *g*) at 4 °C for 10 min. The supernatant was transferred into a new centrifuge tube (2-mL) and mixed with 0.2 mL 25% metaphosphoric acid. After 30 min at 4 °C, the tube was centrifuged (12,000 × *g*) again at 4 °C for 10 min. To quantify SCFAs, a calibration curve for SCFAs was constructed.

### Untargeted metabolomics profiling

Plasma metabolites were extracted as previously described [20]. Metabolomics analysis of plasma sample was performed by the liquid chromatography-mass spectrometry (LC-MS) system followed machine orders. First, all chromatographic separations were performed using an ultra-performance liquid chromatography system (Waters, Milford, MA, USA). For the reversed phase separation, plasma sample used an ACQUITY UPLC BEH C18 column (2.1 mm ×100 mm, 1.7 μm) and liver tissue sample used an ACQUITY UPLC HSS T3 column (2.1 mm × 100 mm, 1.8 μm). A high-resolution tandem mass spectrometer Xevo G2 XS QTOF (Waters) was used to detect metabolites eluted from the column. The Q-TOF was operated in both positive and negative ion modes. In order to test the stability of the LC-MS during the whole acquisition, a quality control sample (Pool of all samples) was gained after every 10 samples. Peak extraction is mainly achieved through the commercial software Progenesis QI (version 2.2), including peak alignment, peak extraction, normalization, deconvolution, and compound identification. Based on quality control sample information, local polynomial regression fitting signal correction (Quality control–based robust LOESS signal correction, QC-RSC) is performed on the real sample signal [33].

### SCFAs-targeted absolute quantification metabolomics

The absolute concentrations of SCFAs in ovarian follicular fluid (*n* = 7) were determined using liquid chromatography-tandem mass spectrometry (LC-MS/MS, Waters I-class–AB Sciex QTRAP 6500) with multiple reaction monitoring. This system allows accurate quantification of low abundance metabolites (up to pg level). Briefly, sample preprocess is as follows. 1) Preparation of standard curve: Take 7 kinds of SCFAs mixed standards (acetate, propionate, butyrate, valerate, isobutyrate, and isovalerate) and carry out gradient dilution; 2) Take 20 μL sample and standard curve sample, add 60 μL cold MeOH/ACN (2:1) of standard song, shake for 5min, precipitate at −20 °C for 4 h, centrifuge at 20,000 × *g* at 4°C for 15 min, and take 40 μL supernatant in an EP tube; 3) Derivatization: Add 20 μL 200mmol/L 3-NPH (solvent 50% ACN) and 20 μL 120 mmol/L EDC-6% pyridine mixture (solvent 50% ACN) into the EP tube containing 40 μL supernatant respectively, and place it in a metal bath incubate with shaking at 40 °C for 30 min. Cool to room temperature on ice and centrifuge briefly. Add 80 μL 1000D internal standard (solvent is 10% ACN) to the derivatized EP tube, and mix well; 4) Add 90 μL H_2_O and 90 μL derivatized sample to the filter plate, centrifuge at 3,000 × *g* at 4 °C for 5 min, take 90 μL filtered liquid in a new 96-well plate, and load 10 μL on the LC-MS. In MultiQuant software (SCIEX, USA), the default parameters are used for automatic identification and integration of each MRM transition (ion pair), and manual inspection is assisted. The content of SCFAs (ng/mL) were obtained by substituting the integrated peak area of the target index in the sample into the standard curve.

## Statistical analysis

Experimental data were analyzed by one-way analysis of variance tests, followed by Fisher’s least significant difference and the Duncan multiple comparison test with GraphPad 8.0 software. Results were presented as mean ± SEM. Significance was presented as ^*^P < 0.05, ^**^P < 0.01, and ^***^P < 0.001.

## Results

### Ovary weight and ovarian follicles development between MS and L × Y sows

**Fig. 1:**
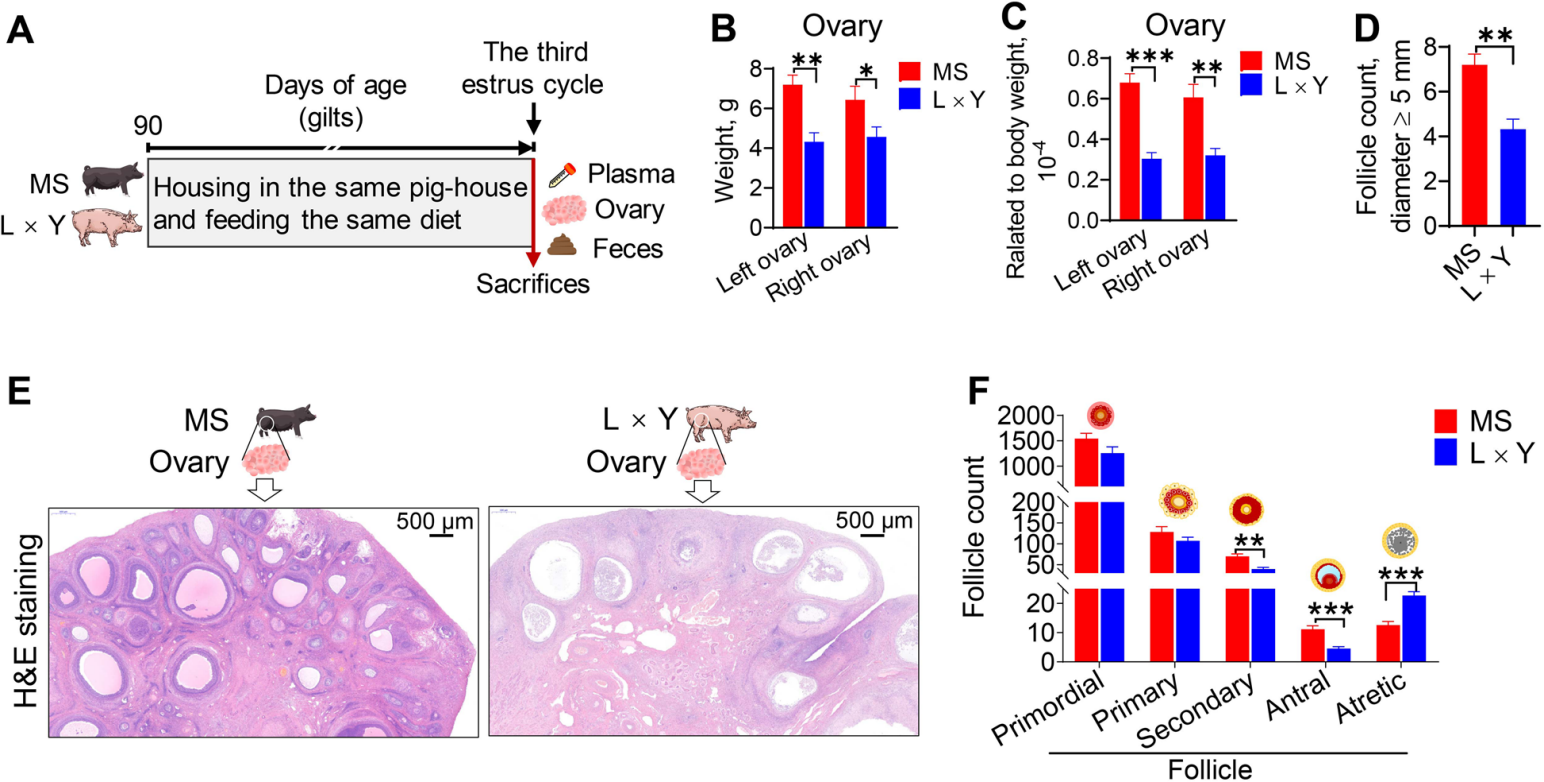
Ovary weight and ovarian follicles development of MS and L × Y sows. **A** Experimental design. **B** Weights of left ovary and right ovary. **C** Relative weights of left ovary and right ovary. **D** The count of follicle with diameter ≥ 5 mm. **E** Representative H&E-stained ovary sections. Scale bar, 500 μm. **F** The follicle counts at different development levels, including primordial, primary, secondary, antral, and atretic follicles. ^*^P < 0.05, ^**^P < 0.01, ^***^P < 0.001, data were shown as means ± SEM; *n* = 7

To determine the role of gut microbiota-ovary axis on follicular development in sows, MS and L × Y sows were housed in the same pig-house and fed with the same diet until the third estrus cycle (Fig. 1A). The absolute and relative ovary weights including the left and right of MS sows were greater than those of L × Y sows (*P* < 0.05) (Fig. 1B and C). MS sows have a greater count of the follicles with diameter ≥ 5 mm (*P* < 0.01) (Fig. 1D). Further morphological analysis of H&E-stained ovary sections showed that MS sows have greater counts of secondary (*P* < 0.01) and antral follicles (*P* < 0.001), but have a smaller count of atretic follicle (*P* < 0.001) (Fig. 1F).

### The apoptosis level of ovarian granulosa cells between MS and L × Y sows

**Fig. 2:**
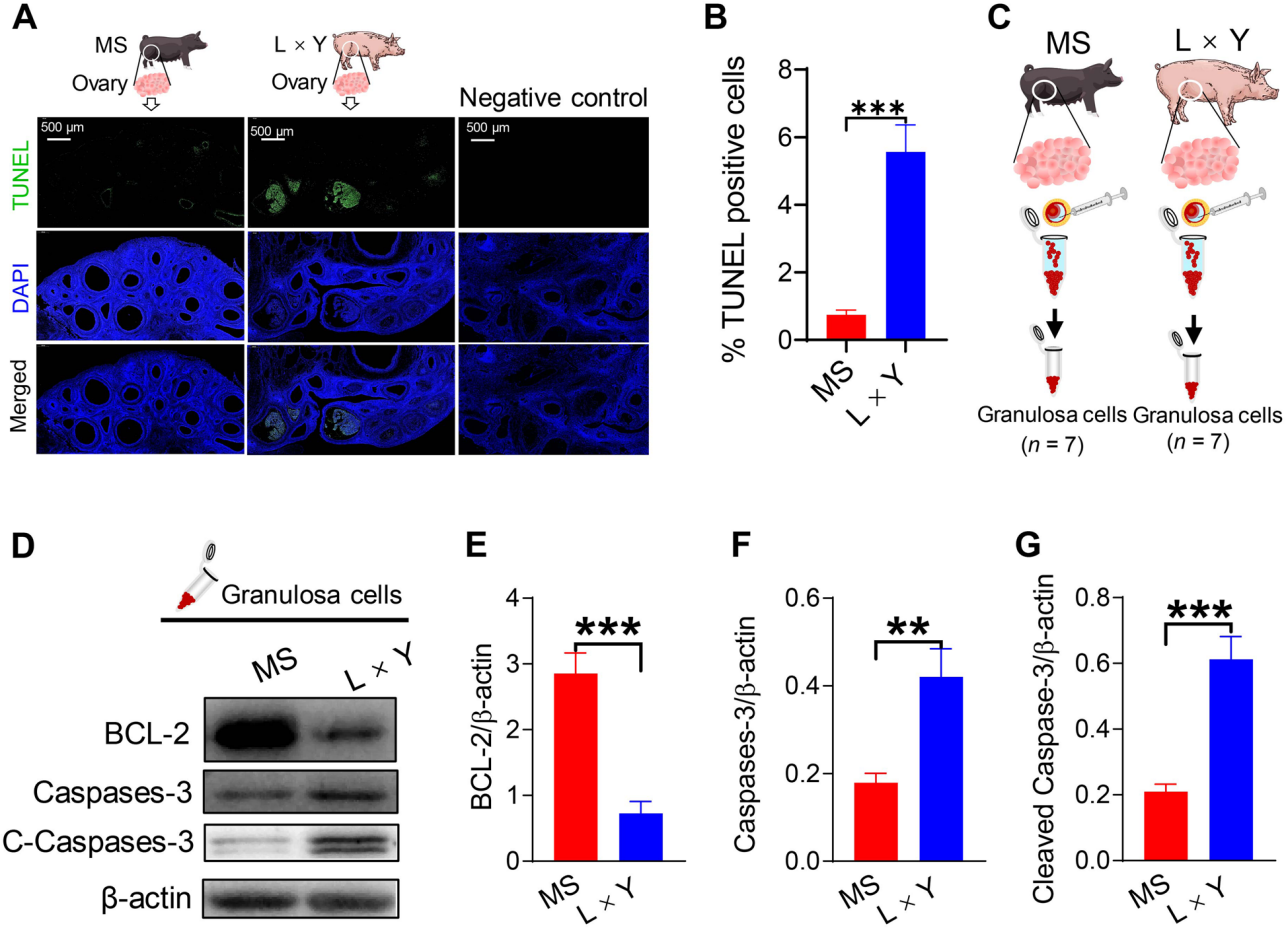
Comparison of ovarian granulosa cells apoptosis between MS and L × Y sows. **A** Representative TUNEL-stained ovary sections. Scale bar, 500 μm. **B** The percentage of TUNEL-positive granulosa cells relative to the DAPI-marked granulosa cells. **C** Collection of granulosa cells from the ovarian follicles. **D**–**G** Western blots of apoptosis-related proteins BCL-2, caspases-3, and cleaved caspases-3 levels related to β-actin in ovarian granulosa cells. ^*^P < 0.05, ^**^P < 0.01, ^***^P < 0.001, data were shown as means ± SEM; *n* = 7

TUNEL-stained ovary sections showed that granulosa cells from MS sows have fewer TUNEL-positive signal than from L × Y sows (*P* < 0.001) (Fig. 2A and B). In the negative control group, false positive reactivity was not observed. Correspondingly, ovarian granulosa cells of MS sows have higher level of anti-apoptosis protein BCL-2 (*P* < 0.001), whereas have lower levels of pro-apoptosis proteins caspases-3 (*P* < 0.01) and cleaved caspases-3 (*P* < 0.001) than those of L × Y sows (Fig. 2C–G).

### The concentrations of hormones in ovarian follicular fluid between MS and L × Y sows

**Fig. 3:**
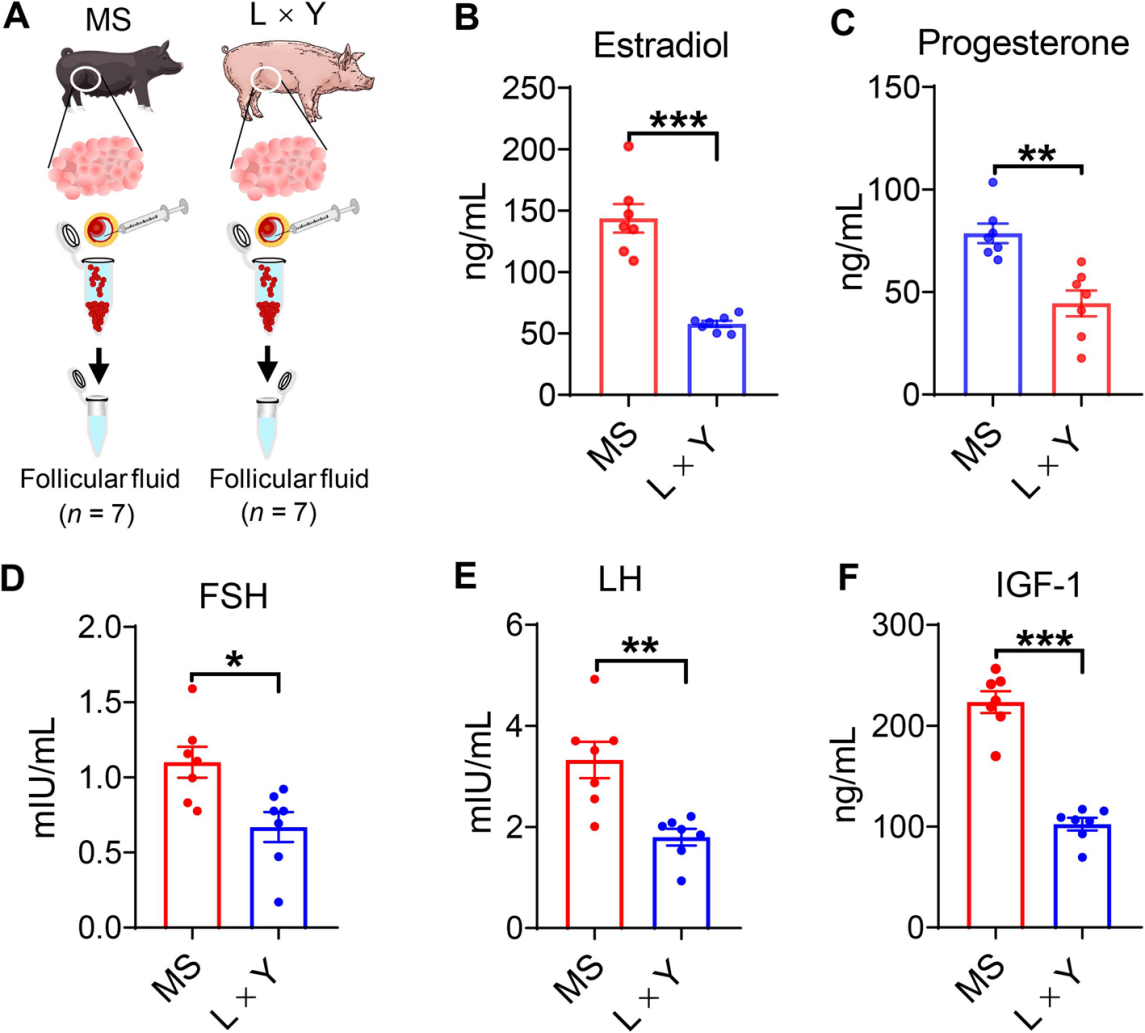
Comparison of the concentrations of hormones in ovarian follicular fluid between MS and L × Y sows. **A** Collection of follicular fluid from the ovarian follicles. The concentrations of estradiol (**B**), progesterone (**C**), FSH (**D**), LH (**E**), and IGF-1 (**F**) in follicular fluid. ^*^P < 0.05, ^**^P < 0.01, ^***^P < 0.001, data were shown as means ± SEM; *n* = 7

Follicular fluid was collected from fresh ovaries (Fig. 3A). The ovarian follicular fluid of MS sows has higher concentrations of estradiol (*P* < 0.001, Fig. 3B), progesterone (*P* < 0.01, Fig. 3C), FSH (*P* < 0.05, Fig. 3D), LH (*P* < 0.01, Fig. 3E), and IGF-1 (*P* < 0.001, Fig. 3F) than L × Y sows.

### The diversity and structure of gut microbiota between MS and L × Y sows

**Fig. 4:**
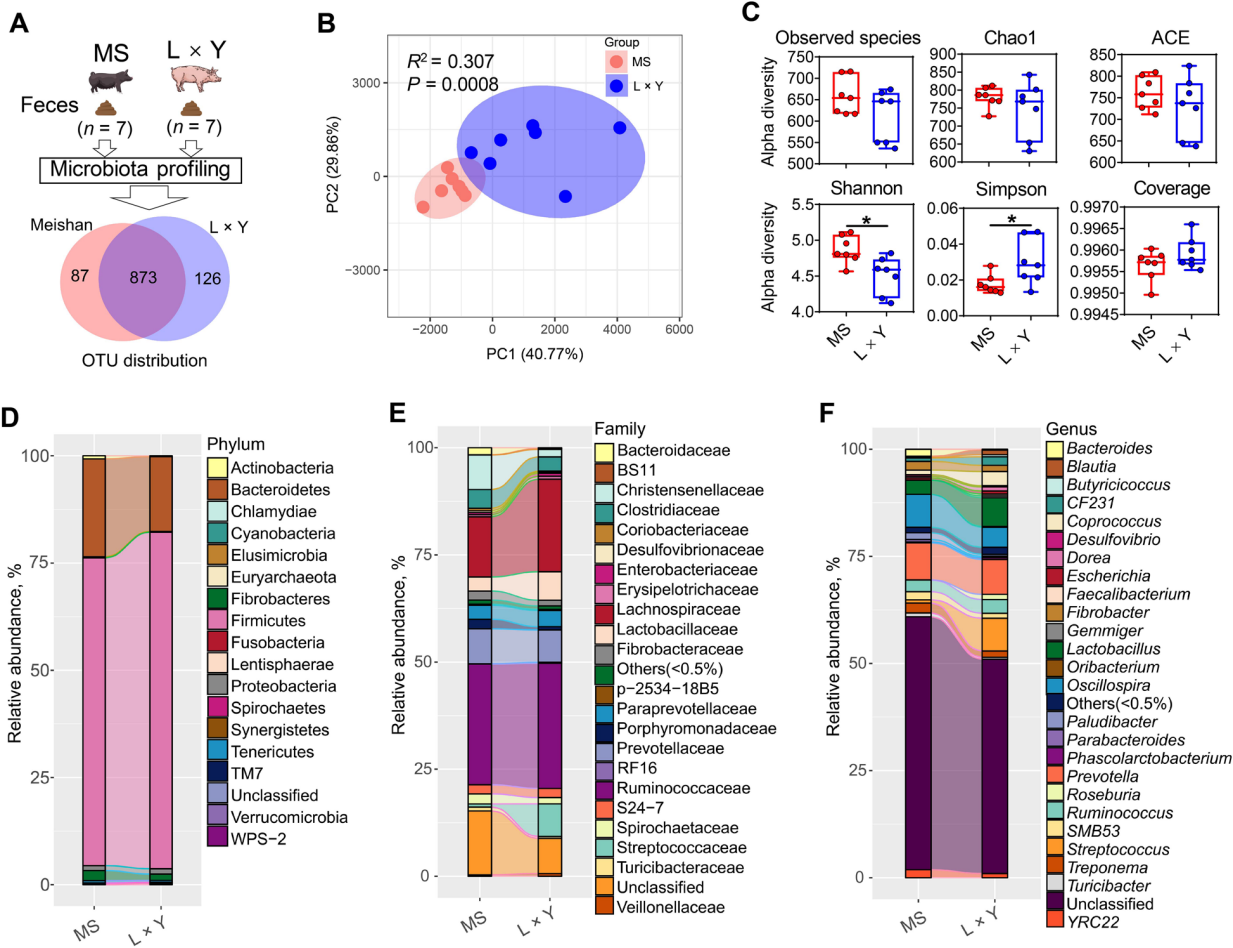
Comparison of the diversity and structure of gut microbiota between MS and L × Y sows. **A** Experimental design and OTU distribution between MS and L × Y sows. **B** Beta diversity presented by PCoA based on weighted UniFrac distanc. Each data point represents a sample (percent variation explained by each PCoA is shown in parentheses). **C** Alpha diversity indices of gut microbiota including Observed species, Chao1, ACE, Shannon, Simpson, and Good coverage, respectively. Linked-bar plots of the relative abundances of microbes at the levels of Phylum (**D**), Family (**E**), and Genus (**F**), respectively. ^*^P < 0.05, ^**^P < 0.01, ^***^P < 0.001, data were shown as means ± SEM; *n* = 7

Gut microbiota analysis of rectum feces showed that there are 87 unique OTUs belong to MS sows, 126 unique OTUs belong to L × Y sows, and 873 OTUs shared in both MS and L × Y sows (Fig. 4A). Beta diversity presented by PCoA showed MS sows formed a distinct cluster markedly away from L × Y sows (*P* = 0.0008, PC1 and PC2 explained 40.77% and 29.86% of the variation, respectively) (Fig. 4B). Compared with L × Y sows, MS sows have improved alpha diversity of gut microbiota including increased Shannon (*P* < 0.05) and decreased Simpson (*P* < 0.05). Linked-bar plots showed that MS sows have an obvious different composition of gut microbiota at the levels of phylum (Fig. 4D), family (Fig. 4E), and genus (Fig. 4F), respectively.

### The marker bacteria, function, and metabolites of gut microbiota between MS and L × Y sows

**Fig. 5:**
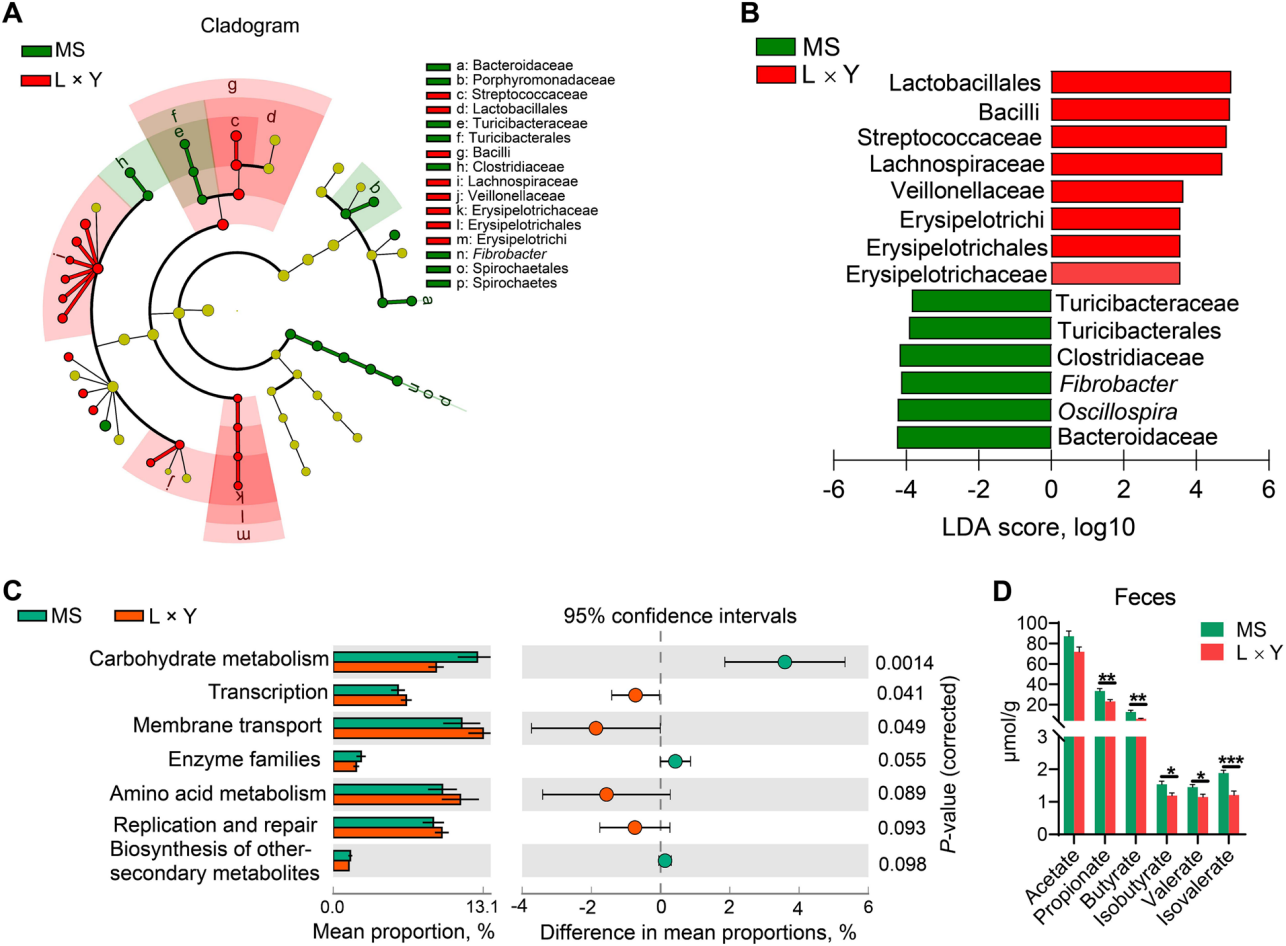
The marker bacteria, function, and metabolites of gut microbiota between MS and L × Y sows. **A** Cladogram using LEfSe showing the phylogenetic distribution of gut microbiota in sows between MS and L × Y sows. **B** Histogram of the LDA score reveals the most differentially abundant taxa between MS and L × Y sows. **C** Extended error bar plot indicating the mean proportion (%) of predicted functions of gut microbiota between MS and L × Y sows. **D** The concentrations of SCFAs including acetate, propionate, butyrate, valerate, isobutyrate and isovalerate in rectal feces. ^*^P < 0.05, ^**^P < 0.01, ^***^P < 0.001, data were shown as means ± SEM; *n* = 7

As shown in Fig. 5A and B, cladogram of LEfSe and histogram of LDA score showed that Turicibacteraceae, Turicibacterales, Clostridiaceae, *Fibrobacter*, *Oscillospira*, and Bacteroidaceae were the marker bacteria of MS sows; Erysipelotrichaceae, Erysipelotrichales, Erysipelotrichi, Veillonellaceae, Lachnospiraceae, Streptococcaceae, Bacilli, and Lactobacillales were the marker bacteria of L × Y sows. Compared with the L × Y sows, gut microbiota of MS sows has an increased function of carbohydrate metabolism (*P* = 0.001) but have decreased functions of translation (*P* = 0.041) and membrane transport *(P* = 0.049) (Fig. 5C). Correspondingly, MS sows have increased concentrations of SCFAs including propionate (*P* < 0.01), butyrate (*P* < 0.01), isobutyrate (*P* < 0.05), valerate (*P* < 0.05), and isovalerate (*P* < 0.001) in rectal feces than L × Y sows (Fig. 5D).

### Metabolomic profiling of plasma between MS and L × Y sows

**Fig. 6:**
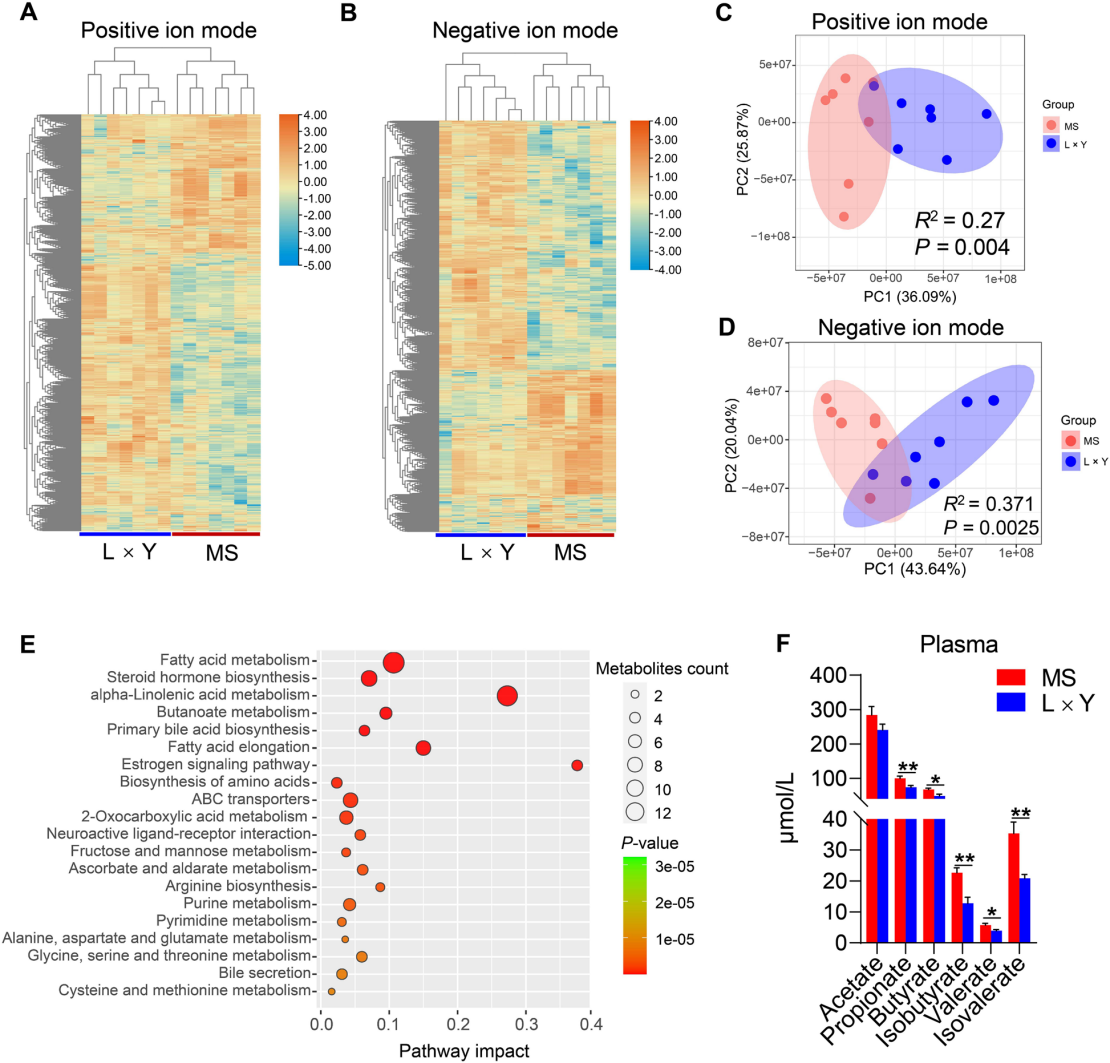
Metabolomic profiling of plasma in MS and L × Y sows. **A**–**B** Heat maps of ion signal intensity including positive and negative ion modes, respectively. **C**–**D** PCA plots based on the ion signal intensity of positive and negative ion modes, respectively. **E** KEGG pathway enrichment analysis for the different metabolites in plasma. **F** The concentrations of SCFAs including acetate, propionate, butyrate, valerate, isobutyrate and isovalerate in plasma. ^*^P < 0.05, ^**^P < 0.01, ^***^P < 0.001, data were shown as means ± SEM; *n* = 7

Heatmaps of untargeted metabolomic profiling showed that MS sows had an obviously different plasma metabolomic profiling compared with L × Y sows (Fig. 6A and B). PCA plots based on the ion signal intensity showed that the plasma metabolomic profiling of MS sows was differed significantly from those of L × Y sows, including positive ion mode (*P* = 0.004, Fig. 6C) and negative ion mode (*P* = 0.0025, Fig. 6D), respectively. As shown in Fig. 6E, these different metabolites in plasma enriched in pathways of fatty acid metabolism, steroid hormone biosynthesis, alpha-linolenic acid metabolism, butanoate metabolism, primary bile acid biosynthesis, fatty acid elongation, estrogen signaling pathway, biosynthesis of amino acids, ABC transporters, and 2-oxocarboxylic acid metabolism, etc. Correspondingly, MS sows have increased concentrations of plasma SCFAs including propionate (*P* < 0.01), butyrate (*P* < 0.05), isobutyrate (*P* < 0.05), valerate (*P* < 0.05), and isovalerate (*P* < 0.01) than L × Y sows (Fig. 6F).

### Correlation analysis among gut microbiome and follicular development indices

**Fig. 7:**
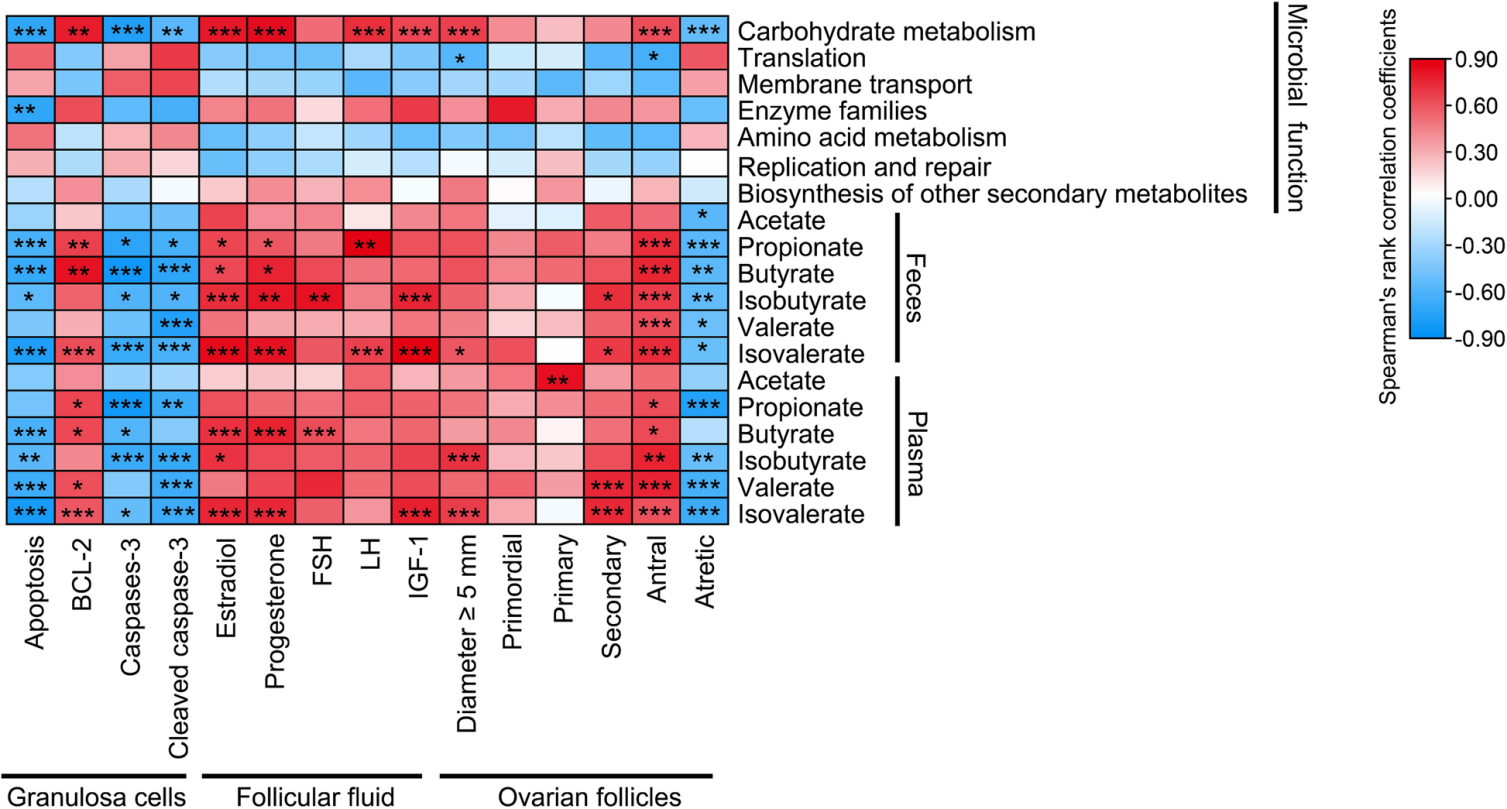
Correlation analysis among gut microbiome and follicular development indices. Heat map of the spearman’s rank correlation coefficient among the gut microbiota and follicular development indices. In the figure, the blue color represents the negative correlation, while the red color represents a positive correlation. ^*^P < 0.05, ^**^P < 0.01, ^***^P < 0.001, data were shown as means ± SEM; *n* = 7

As shown in Fig. 7, the differential gut microbial function were correlated with follicular development indices, especially carbohydrate metabolism was significant (*P* < 0.01) positively associated with follicles of diameter ≥ 5 mm, antral follicles, estradiol, progesterone, LH, IGF-1, and BCL-2; but was negatively associated with apoptosis, caspases-3, and cleaved caspases-3, respectively. Correspondingly, for SCFAs in feces, acetate was negatively associated with atretic follicles (*P* < 0.05); propionate was significant (*P* < 0.05) positively associated with antral follicles, LH, progesterone, estradiol, and BCL-2, but was significant (*P* < 0.05) negatively associated with atretic follicles, cleaved caspases-3, caspases-3, and apoptosis; butyrate was significant (*P* < 0.05) positively associated with antral follicles, progesterone, estradiol, and BCL-2, but was significant (*P* < 0.05) negatively associated with atretic follicles, cleaved caspases-3, caspases-3, and apoptosis; isobutyrate was significant (*P* < 0.05) positively associated with antral follicles, secondary follicles, IGF-1, FSH, progesterone, and estradiol, but was significant (*P* < 0.05) negatively associated with atretic follicles, cleaved caspases-3, caspases-3, and apoptosis; valerate was significant (*P* < 0.001) positively associated with antral follicles, but was significant (*P* < 0.05) negatively associated with atretic follicles and cleaved caspases-3; isovalerate was significant (*P* < 0.05) positively associated with antral follicles, secondary follicles, follicles of diameter ≥ 5 mm, IGF-1, LH, progesterone, estradiol, and BCL-2, but was significant (*P* < 0.05) negatively associated with atretic follicles, cleaved caspases-3, caspases-3, and apoptosis. For SCFAs in plasma, acetate was significant (*P* < 0.01) positively associated with primary follicles; propionate was significant (*P* < 0.05) positively associated with antral follicles and BCL-2, but was significant (*P* < 0.05) negatively associated with atretic follicles, cleaved caspases-3, and caspases-3; butyrate was significant (*P* < 0.05) positively associated with antral follicles, FSH, progesterone, estradiol, and BCL-2, but was significant (*P* < 0.05) negatively associated with caspases-3, and apoptosis; isobutyrate was significant (*P* < 0.05) positively associated with antral follicles, follicles of diameter ≥ 5 mm, and estradiol, but was significant (*P* < 0.05) negatively associated with atretic follicles, cleaved caspases-3, caspases-3, and apoptosis; valerate was significant (*P* < 0.001) positively associated with antral follicles, secondary follicles, and BCL-2, but was significant (*P* < 0.05) negatively associated with atretic follicles, cleaved caspases-3, and apoptosis; isovalerate was significant (*P* < 0.001) positively associated with antral follicles, secondary follicles, follicles of diameter ≥ 5 mm, IGF-1, progesterone, estradiol, and BCL-2, but was significant (*P* < 0.05) negatively associated with atretic follicles, cleaved caspases-3, caspases-3, and apoptosis.

### Effects of physiological concentration of SCFAs on granulosa cells apoptosis in vitro

**Fig. 8:**
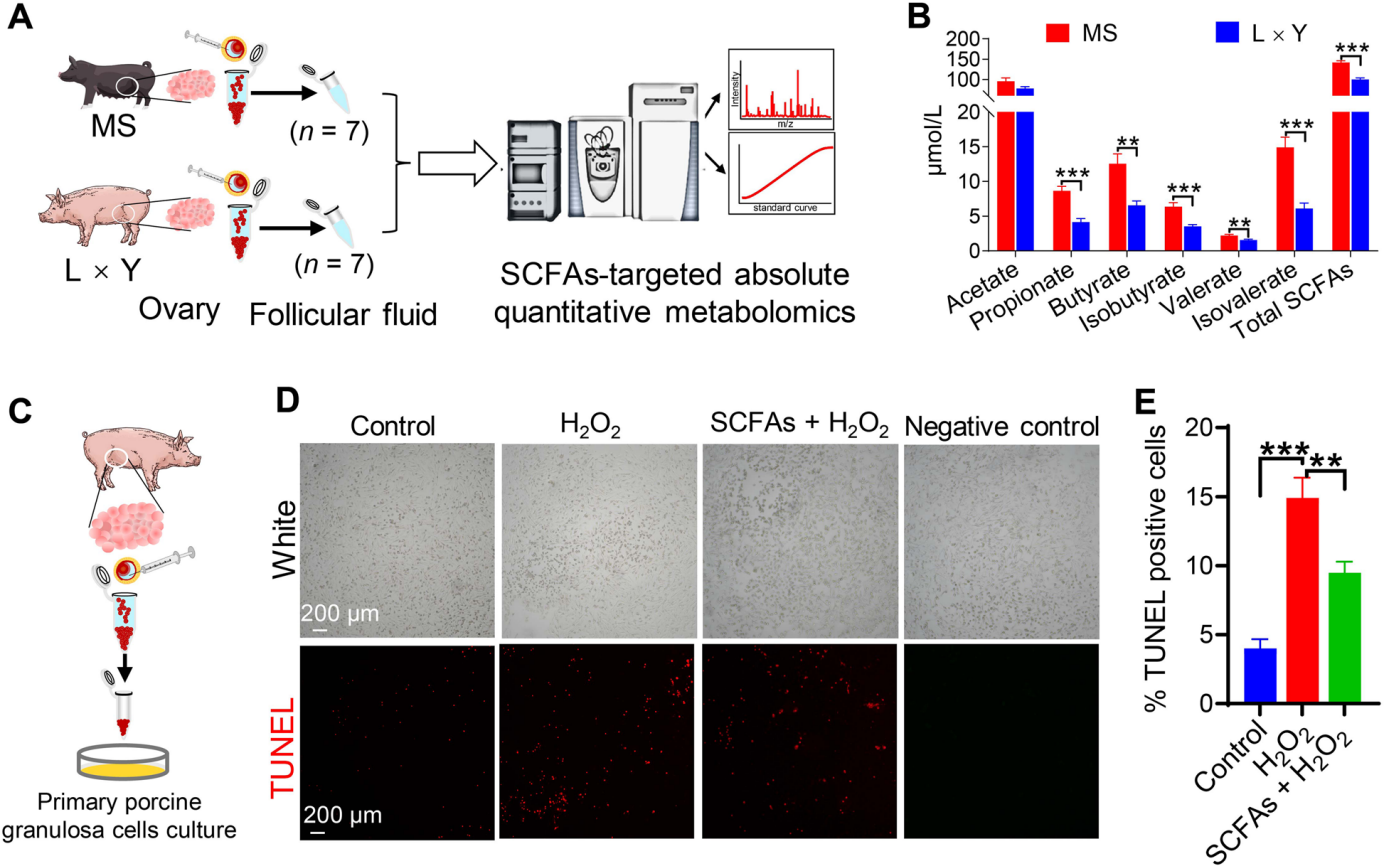
Determination of SCFAs physiological concentrations in follicular fluid and their effects on H_2_O_2_-induced poricine granulosa cells apoptosis in vitro. **A** SCFAs-targeted absolute quantitative metabolomics analysis of ovarian follicular fluid in MS and L × Y sows, respectively. **B** The concentrations of SCFAs including acetate, propionate, butyrate, valerate, isobutyrate, isovalerate, and total SCFAs in ovarian follicular fluid of MS and L × Y sows, respectively. **C** Collecting and culture of porcine ovarian granulosa cells in vitro. **D** Representative white light and TUNEL-stained pictures of porcine ovarian granulosa cells treated with vehicle, or H_2_O_2_, or H_2_O_2_ + SCFAs, respectively. Scale bar, 200 μm. **E** The percentage of TUNEL-positive porcine granulosa cells. ^*^P < 0.05, ^**^P < 0.01, ^***^P < 0.001, data were shown as means ± SEM; *n* = 7

As shown in Fig. 8A, SCFAs-targeted absolute quantitative metabolomics analysis was conducted to determine the physiological concentrations of SCFAs in the ovarian follicular fluid collected from fresh ovarian follicles of MS and L × Y sows, respectively. The results of SCFAs-targeted absolute quantitative metabolomics showed that ovarian follicular fluid of MS sows had increased concentrations of propionate (*P* < 0.001), butyrate (*P* < 0.01), isobutyrate (*P* < 0.001), valerate (*P* < 0.01), isovalerate (*P* < 0.001), and total SCFAs (*P* < 0.001) than L × Y sows (Fig. 8B). Porcine ovarian granulosa cells were collected from fresh ovaries and then cultured in vitro (Fig. 8C). Fig. 8D and E showed that H_2_O_2_ treatment markedly increased apoptosis of porcine ovarian granulosa cells compared with the Ctrl (*P* < 0.001), which was ameliorated by SCFAs treatment at the concentrations in follicular fluid (*P* < 0.01). In the negative control group, false positive reactivity was not observed.

## Discussion

Here, we found that MS sows have greater ovary weight and have more follicles with diameter ≥ 5 mm than L × Y sows. Further morphological analysis indicated that MS sows have more secondary and antral follicles but have less atretic follicle than L × Y sows. These results are comparable with an earlier comparative study which showed that hyper-fertility MS sows even had 11.3 more pre-ovulatory follicles and ovulate 10 more occytes in an estrus cycle than Large-White hybrid sows [5]. Ovarian granulosa cell apoptosis-induced follicular atresia results in over 99% of the follicles undergo atretic degeneration before ovulation [3, 4]. For the first time, we demonstrated MS sows had significantly fewer apoptotic granulosa cells in antral follicles than L × Y sows with TUNEL-staining in situ. This extraordinary phenotype was further supported by the attenuated apoptosis signal pathway (increased BCL-2, decreased caspases-3 and cleaved caspases-3) of ovarian granulosa cell in MS sows than in L × Y sows. In sows, ovarian granulosa cells can produce steroid hormones (estradiol and progesterone) and provide an appropriate microenvironment for oocytes to meiosis and maturation [19, 34, 35]. Given the determinative role of granulosa cells in follicular development [34, 35], the high-quality granulosa cells may account for the hyper-ovulation rate of MS sows. Granulosa cells in antral follicles synthesize plenty of FSH receptors and LH receptors on each cell, making them extremely sensitive to FSH and LH [35]. Correspondingly, we found MS sows had increased concentrations of estradiol, progesterone, FSH, LH, and IGF-1 in ovarian follicular fluid than L × Y sows. Given that granulosa cells produce estradiol [34, 35], the reduced apoptotic granulosa cells may account for the higher estradiol concentrations in MS follicular fluid than in hybrid Large White pigs [5]. In addition, FSH together with LH can stimulate granulosa cells synthesize estradiol and progesterone, and also coordinate follicular maturation and ovulation [36]. On the other hand, IGF-1 can synergize with FSH to stimulate granulosa cell proliferation and steroidogenesis [37]. Plasma concentrations of both FSH and LH are also positively associated with the ovulation rate in crossbreed sows [38]. Taken the above evidence together, high-quality ovarian granulosa cells may account for the superiority of MS sows in follicular development and ovulation rate than L × Y sows although the underlying mechanism remains unclear.

Gut microbiota, known as the second genome and a substantial endocrine organ, has been reported to affect host reproductive hormones production and metabolism [7, 39–43]. A recent study that recruited 18 fertile female subjects and 18 patients with infertility matched by age showed marked differences in the abundance of gut microbiota between fertile and infertile females [44]. Moreover, partially hydrolyzed guar gum supplementation improved gut microbial dysbiosis and the success of pregnancy in females with infertility [44]. Jiang et al. [16] found that fecal progesterone and estradiol concentrations were increased in MS sows whose litter sizes and gut microbiota had been improved by dietary crude fiber supplementation. Hence, it could be speculated that gut microbiota may contribute to the differences of follicular hormone concentrations between MS and L × Y sows. Under the same feed and housing conditions, MS sows developed a distinctive gut microbiota of improved structure and diversity than L × Y sows. Turicibacteraceae, Turicibacterales, Clostridiaceae, *Fibrobacter*, *Oscillospira*, and Bacteroidaceae were the marker bacteria of MS sows, whereas Erysipelotrichaceae, Erysipelotrichales, Erysipelotrichi, Veillonellaceae, Lachnospiraceae, Streptococcaceae, Bacilli ,and Lactobacillales were the marker bacteria of L × Y sows. Compared with the L × Y sows, gut microbiota of MS sows has an increased function of carbohydrate metabolism but have decreased functions of translation and membrane transport. Correspondingly, MS sows have increased concentrations of SCFAs including propionate, butyrate, isobutyrate, valerate, and isovalerate in rectal feces than L × Y sows. Compared well with our results, the alpha diversity of gut microbiota, the relative abundance of *Fibrobacter*, and fecal SCFAs were increased in MS sows whose litter sizes had been improved by dietary crude fiber supplementation [16].

To explore the connection between the gut microbiota and the distal ovarian follicular development, we profiled the plasma metabolome features using untargeted metabolomic and demonstrated a markedly distinguishing plasma metabolomics profiling between MS and L × Y sows. These different metabolites in plasma enriched in pathways of fatty acid metabolism, steroid hormone biosynthesis, alpha-linolenic acid metabolism, butanoate metabolism, primary bile acid biosynthesis, fatty acid elongation, estrogen signaling pathway, biosynthesis of amino acids, ABC transporters, and 2-Oxocarboxylic acid metabolism, etc. Correspondingly, MS sows have increased concentrations of plasma SCFAs including propionate, butyrate, isobutyrate, valerate, and isovalerate than L × Y sows. In addition, correlation analysis showed that the concentrations of SCFAs in both feces and plasma were significant negatively associated with the apoptosis of ovarian granulosa cells, indicating that the increased SCFAs may contribute to the porcine granulosa cells apoptosis amelioration observed in MS sows. In proximal tissues such as the intestine, adipose, and pancreas, SCFAs can regulate host immunity, inflammation, and metabolism by activating G protein-coupled receptors (GPCRs) [45]. However, the activation of GPCRs by SCFAs is receptor-preferred and concentration-dependent [46]. In vivo, butyrate regulates porcine granulosa cells to secrete progesterone and estradiol via cAMP signaling pathway in a dose-dependent manner [47]. The effective dose of butyrate on the secretion of progesterone was over 0.05 mmol/L, and the effective dose for estradiol secretion was over 5 mmol/L [47]. These evidences indicated that the in vitro levels of SCFAs may not fully reflect the physiological levels and function in vivo [47]. Earlier study using proton nuclear magnetic resonance spectroscopy showed that the acetate concentrations were 0.34–1.5 mmol/L in ovarian follicular fluids of pig [48]. However, to our knowledge, the physiological concentrations of the others SCFAs in porcine ovarian follicular fluid remain unreported because of the concentrations are too low to be determined using a conventional method [14, 47]. Targeted metabolomics has a pg-level sensitivity for accurate quantification of low-abundance metabolites. With targeted bile acid metabolomics, Gu et al. [49] determined bile acids in human plasma at the nmol/L level. In this study, with SCFAs-targeted absolute quantitative metabolomics, for the first time, we reported that ovarian follicular fluid of MS sows had higher SCFAs concentrations than those of L × Y sows including propionate (8.65 ± 1.55 vs. 4.15 ± 1.29 μmol/L), butyrate (12.56 ± 3.43 vs. 6.56 ± 1.57 μmol/L), isobutyrate (6.38 ± 1.34 vs. 3.54 ± 0.63 μmol/L), valerate (2.21 ± 0.42 vs. 1.56 ± 0.29 μmol/L), isovalerate(14.91 ± 3.60 vs. 6.11 ± 1.90 μmol/L), and total SCFAs (140.42 ± 30.50 vs. 100.00 ± 16.50 μmol/L), respectively. Inconsistent with Gosden et al., we found that the acetate concentration in ovarian follicular fluid was 86.91 ± 18.42 μmol/L, which was markedly lower than 0.34–1.5 mmol/L [48]. This variation may be because of the different detection method. Given that the effect of SCFAs is concentration-dependent [46, 47], we determined whether the SCFAs were sufficient to ameliorate porcine granulosa cells apoptosis in vitro. Not surprisingly, these physiological concentrations of SCFAs in porcine ovarian follicular fluid could ameliorate H_2_O_2_-induced porcine granulosa cells apoptosis in vitro. Evidence in vitro suggested that acetate and propionate can activate GPR43 at low concentration (1 mmol/L) to inhibit the apoptosis of cultured cells in vitro; while at high concentration (10 mmol/L), they promote the apoptosis of colorectal cancer cells [50, 51]. In vitro experiments showed that butyric acid could promote the expression of GPCRs genes in porcine ovarian granulosa cells only when it reached 10 mmol/L [47]. It is generally believed that the physiological concentration of SCFAs in porcine follicles cannot reach the threshold of activating GPCRs [14, 52]. In this study, the physiological concentrations of SCFAs in porcine ovarian follicular fluid are also not reach the threshold of activating GPCRs [14, 52]. Considering SCFAs share the GPCRs and have competitive antagonistic/additive relationship against GPCRs [45], the role of GPCRs in SCFAs ameliorating porcine granulosa cells apoptosis remains uncertain. On the other hand, recent studies have shown that affecting the epigenetic modification of cells in the intestine and even the distant organs is a new mechanism by which gut microbial metabolites SCFAs can also regulate biological processes such as cell cycle, cell proliferation, and apoptosis [45, 53–56]. Such as SCFAs can regulate histone acetylation modification by providing acetylation substrates or affecting the activities of histone deacetylases and histone acetylases, thereby regulating cell physiology [45]. In addition, SCFAs have the potential to affect novel modifications of histones-propionylation and butyrylation [54, 56]. Although the mechanism by which physiological concentrations of SCFAs ameliorates porcine granulosa cells apoptosis is complicated and remains to be identified, our results suggested that modulating gut microbiota to produce SCFAs or supplementation with SCFAs directly may be a novel approach to ameliorate ovarian dysfunction induced by granulosa cells apoptosis. For example, accumulating evidence indicated that high-energy diets could decrease oocyte quality and ovarian function, increase granulosa cells apoptosis and follicular atresia, thus potentially decreasing reproductive performance and shortening the reproductive span [14, 57, 58]. For sows, Zhuo et al. [59] found that high-fat (high-energy) diets could increase granulosa cells apoptosis and follicular atresia, reduce SCFAs-producing gut microbes, whereas dietary fibre supplementation restored this loss and increased the relative abundance of representative SCFAs-producing gut microbes. The authors suggested that dietary fibre supplementation protected against high fat feeding-induced granulosa cells apoptosis and follicular atresia in an indirectly approach via gut microbiota-SCFAs-serotonin/melatonin-ovary axis [59]. On the other hand, according to our results, dietary fibre supplementation may decrease high fat feeding-induced follicular atresia directly by gut microbiota-derived SCFAs ameliorating ovarian granulosa cells apoptosis in sows. Overall, these results provide insight into preventing granulosa cells apoptosis-related ovarian dysfunction.

## Conclusions

This study provides insight into the regulating effects of gut microbiota-ovary axis on follicular development in sows by a comprehensively comparative study between hyper-ovulation rate MS pigs and normal-ovulation rate L × Y pigs. The high levels of estradiol, progesterone, FSH, LH, and IGF-1, together with the less apoptotic granulosa cells in ovarian follicles may account for the hyper-ovulation rate of MS pigs. MS pigs have higher SCFAs concentrations in porcine ovarian follicular fluid, which may be because of their enhanced carbohydrate metabolism function of gut microbiota than L × Y pigs. These physiological concentrations of SCFAs in porcine ovarian follicular fluid of MS pigs could ameliorate porcine granulosa cells apoptosis in vitro. Therefore, gut microbiota participate in regulating ovarian follicular development via SCFAs affecting granulosa cells apoptosis in sows.

## Supplementary Information

**Additional file1:** **Table S1. **Ingredients and nutrients composition of the diet used in this trial.

## Data Availability

All data generated or analyzed during this study can be made available by the corresponding author upon reasonable request.

## Abbreviations

BCL-2: B-cell lymphoma 2
ELISA: Enzyme-linked immunosorbent assay
FSH: Follicle-stimulating hormone
GPCRs: G protein-coupled receptors
H&E: Hematoxylin and eosin
IGF-1: Insulin-like growth factor 1
LC-MS: Liquid chromatography-mass spectrometry
LDA: Linear discriminant analysis
LEfSe: Linear discriminant analysis effect size analysis
LH: Luteinizing hormone
L × Y: Landrace × Yorkshire
MS: Meishan
OTU: Operational taxonomic units
PCoA: Principal co-ordinates analysis
PICRUSt2: Phylogenetic Investigation of Communities by Reconstruction of Unobserved States 2
SCFAs: Short chain fatty acids
TUNEL: Terminal terminal-deoxynucleoitidyl transferase mediated nick end labeling
